# Characterization of the CBM50 Gene Family in *Tilletia horrida* and Identification of the Putative Effector Gene ThCBM50_1

**DOI:** 10.3390/jof10120856

**Published:** 2024-12-11

**Authors:** Ting Xiang, Deze Xu, Linxiu Pan, Dongyu Zhai, Yu Zhang, Aiping Zheng, Desuo Yin, Aijun Wang

**Affiliations:** 1 College of Agronomy, Guangxi University, Nanning 530004, China; 2 College of Agronomy, Sichuan Agricultural University, Chengdu 610065, China; 3 Food Crop Research Institute, Hubei Academy of Agriculture Sciences, Wuhan 430023, China; 4 College of Plant Protection, Henan Agricultural University, Zhengzhou 450046, China

**Keywords:** *Tilletia horrida*, rice, CBM50s, pathogenicity

## Abstract

Carbohydrate-binding modules (CBMs) are essential virulence factors in phytopathogens, particularly the extensively studied members from the CBM50 gene family, which are known as lysin motif (LysM) effectors and which play crucial roles in plant–pathogen interactions. However, the function of CBM50 in *Tilletia horrida* has yet to be fully studied. In this study, we identified seven CBM50 genes from the *T. horrida* genome through complete sequence analysis and functional annotation. Their phylogenetic relationships, conserved motifs, promoter elements, and expression profile were further analyzed. The phylogenetic analysis indicated that these seven ThCBM50 genes were divided into three groups, and close associations were observed among proteins with similar protein motifs. The promoter cis-acting elements analysis revealed that these ThCBM50 proteins may be involved in the regulation of the phytohormones, stress response, and meristem expression of the host plant during *T. horrida* infection. The transcriptome data indicated that four ThCBM50 genes were upregulated during *T. horrida* infection. We further found that *ThCBM50_1* caused cell death in the leaves of *Nicotiana benthamiana*, and its signal peptide (SP) had a secreting function. These results offer important clues that highlight the features of *T. horrida* CBM50 family proteins and set the stage for further investigation into their roles in the interactions between *T. horrida* and rice.

## 1. Introduction

Carbohydrate activity enzymes (CAZymes) secreted by plant pathogenic fungi can degrade the polysaccharide materials in plant cell walls, thus facilitating infection and gaining nutrition [1]. Carbohydrate-binding modules (CBMs) play a role in the degradation of insoluble polysaccharides through appendage to CAZymes [2]. Based on their sequence homology, CBMs are divided into 87 families in the CAZy database [3]. Considering dozens of plant pathogenic fungi, CBMs have been confirmed to be involved in pathogenicity or virulence [4,5,6]. For instance, the deletion of the CBM gene PcCBP3 results in significantly reduced virulence of *Phytophthora capsica* on the host plant [7].

Members from the CBM 50 (CBM50) gene family are also known as lysin motif (LysM) domains, with an approximate length of 50 amino acids [8]. The functional characterization of CBM50 genes from fungal plant pathogens revealed that CBM50 proteins can serve as effectors and play a role in the suppression of chitin-triggered plant immunity by sequestering the chitin oligosaccharides released from the fungal cell walls, resulting in the promotion of pathogen infection [9,10]. For example, a CBM50 protein from *Cladosporium fulvum*, Ecp6, could suppress chitin-triggered immunity in plants [11]. The CBM50 proteins Mg1LysM and Mg3LysM, belonging to *Zymoseptoria tritici*, have been found to protect fungal hyphae against degradation by plant chitinases and play a crucial role as effectors in the interactions between *Z. tritici* and its host [9]. Furthermore, the CBM50 protein Vd2LysM was shown to be involved in the virulence of *Verticillium dahliae* through the perturbation of the activation of chitin-triggered host immunity [12].

Rice (*Oryza sativa*) is an essential grain crop that is considered a food source for approximately 20% of people worldwide [13,14]. Rice kernel smut (RKS), caused by the biotrophic fungus *Tilletia horrida*, is widely distributed in hybrid rice-producing regions in Africa, America, and Asia [15]. *T. horrida* infects rice floral organs at the flowering stage, manifesting as a dark black powdery mass of spores in the kernels at yellow ripeness [16]. The disease occurrence rate of *T. horrida* can reach 60% under optimum environmental conditions, resulting in more than a 20% decrease in rice yield [17]. Currently, this disease is a major threat to high hybrid rice production in most rice-growing areas. Despite all this, studies on the pathogenic genes of *T. horrida* are scarce, making this key rice disease difficult to manage.

In this study, we characterized the CBM50 gene family of *T. horrida* comprising members in the genome of *T. horrida* JY-521 [18]. The physicochemical properties, conserved motifs, gene structure, and promoter cis-acting elements of these CBM50 proteins were analyzed. The phylogenetic analysis revealed that the seven ThCBM50 members were divided into three groups, and close associations were identified among proteins with similar protein motifs. The promoter cis-acting element analysis indicated that these ThCBM50 members may be involved in the regulation of phytohormones, stress response, and meristem expression. We also observed the expression pattern of these CBM50 genes during *T. horrida* infection according to previously reported transcriptome data [18,19]. Furthermore, we found that *ThCBM50_1* was upregulated during the early stage of *T. horrida* infection and caused cell death in the leaves of *Nicotiana benthamiana*. The predicted signal peptide (SP) of ThCBM50_1 (ThCBM50_1^SP^) exhibited a secreting function. These results contribute to a better understanding of CBM50 functions in the interactions between *T. horrida* and rice.

## 2. Materials and Methods

### 2.1. Identification and Analysis of Physicochemical Properties Analysis of ThCBM50 Family Genes

We used the *T. horrida* JY-521 genomic data and relevant annotation files from a previous report [18]. The CBM50 proteins belonging to the *T. horrida* JY-521 genome were annotated using the CAZy database (http://www.cazy.org/, accessed on 10 September 2024), with an e-value of less than 1 × e^−5^. The conserved domains of candidate ThCBM50 proteins were detected using the NCBI website (https://www.ncbi.nlm.nih.gov/, accessed on 20 September 2024). The annotated CBM50 proteins that contained LysM domains were considered ThCBM50 proteins. The online ExPASy software (https://web.expasy.org/protparam/, accessed on 20 September 2024) was used to analyze the physicochemical properties of the ThCBM50 proteins, such as the aa number, molecular weight, theoretical pI, instability index, and GRAVH [20]. The website https://wolfpsort.hgc.jp/ (accessed on 20 September 2024) was used to predict the subcellular localization of ThCBM50 proteins.

### 2.2. Phylogenetic Analysis

The homologous proteins of ThCBM50s in other smut fungi, namely *T. laevis*, *T. caries*, *T. controversa*, and *T. anomala*, were identified using BLAST in the NCBI website (https://www.ncbi.nlm.nih.gov/, accessed on 21 September 2024). We generated the phylogenetic tree of the 7 ThCBM50 members and their homologous proteins using the MEGA 7.0 software with a neighbor-joining (NJ) method.

### 2.3. Analysis of Gene Structure, Protein Motifs, and Protein Structure

The exons or introns of ThCBM50 genes were detected using GSDS (https://gsds.gao-lab.org/index.php, accessed on 21 September 2024) [21]. The conservative motif of ThCBM50 proteins was analyzed using the MEME website (http://meme-suite.org, accessed on 21 September 2024), with a pattern count of 10 and the other parameters set to default values [22]. The protein secondary structures of ThCBM50s were analyzed using SPOMA [23]. The website https://www.swissmodel.expasy.org/ (accessed on 21 September 2024) was used to predict the protein 3D structures of ThCBM50s [24,25].

### 2.4. Promoter Cis-Acting Regulatory Element Analysis

We extracted the promoter sequences 2000 bp upstream of the transcription start site of the ThCBM50 genes from the *T. horrida* JY-521 genome sequences [18]. The PlantCARE online network server (http://bioinformatics.psb.ugent.be/webtools/plantcare/html/, accessed on 22 September 2024) was used for analyzing the cis-acting regulatory elements of the 7 ThCBM50 genes.

### 2.5. qRT-PCR Analysis

The Bio-Rad CFX96 Real-Time PCR System (Bio-Rad, Foster City, CA, USA) was used for qRT-PCR analysis. The fungal-conserved gene *UBQ* was used as an internal reference gene. The relative expression levels were determined using the 2^−ΔΔCt^ algorithm. Three independent experiments were conducted, with four biological replicates each. Statistical analysis was conducted using a one-way analysis of variance, followed by Tukey’s multiple-comparison test. The primer sequences used in this study are provided in Table S3.

### 2.6. Yeast Secretion Assay Involving TTC

For yeast secretion assays, the ThCBM50_1^SP^ sequence was inserted into a pSUC2 vector encoding the invertase without SP [26] using specific primers (Table S3). Then, the recombinant vector was transformed into the yeast strain YTK12 (without invertase) using the Frozen-EZ Yeast Transformation II Kit (Zymo Research). The transformants were cultured on a CMD-W solid medium (6.7 g yeast N base without amino acids, 0.75 g tryptophan [W] dropout supplement, 20 g sucrose, 1 g glucose, 15 g agar, and 1000 mL distilled water [pH 5.8]) and a YPRAA solid medium (10 g yeast extract, 20 g peptone, 20 g raffinose, 2 μg antimycin A, 15 g agar, and 1000 mL distilled water [pH 5.8]). The invertase activity was further tested using the TTC assay according to previously described methods [27].

### 2.7. Transient Expression Assays

The ThCBM50_1 sequence was inserted into a 35S-PMDC32 vector using specific primers (Table S3). The recombinant vector was transformed into *A. tumefaciens* GV3101. The positive transformants were identified using PCR methods, and then the positive transformants were cultured in an LB medium at 28 °C with shaking at 200 rpm for 48 h. The recombinant positive transformants were collected via high-speed centrifugation and resuspended using MES buffer (200 μM acetosyringone, 10 mM MgCl2, and 10 mM MES [pH 5.6]). The bacterial suspension (OD600 = 0.6) was then agroinfiltrated into *N. benthamiana* leaves. Bacterial suspensions with the BAX protein and GFP were used as positive and negative controls, respectively. The symptoms were detected 4–6 days post-inoculation (dpi).

## 3. Results

### 3.1. Identification and Characterization of ThCBM50 Gene Family

A total of seven CBM50 members (named ThCBM50_1- ThCBM50_7) were identified from the *T. horrida* JY-521 genome (Table 1), and we analyzed their protein length, molecular weight, isoelectric point (pI), instability index, and the grand average of hydropathicity (GRAVH). Their protein length ranged from 286 amino acids (aa) (ThCBM50_5) to 1368 aa (ThCBM50_1), with protein molecular weights ranging from 30.81 kDa to 145.57 kDa, respectively (Table 1). The pI of ThCBM50_1 was the lowest, at 6.24, and the highest belonged to ThCBM50_2, at 10.27. The average pI of these seven ThCBM50 proteins was 7.85, indicating alkalinity (Table 1). These results illustrate that ThCBM50 gene members have a wide range of pI and molecular weights. The instability index of the great majority of these genes was greater than 40, indicating unstable proteins, except for ThCBM50_4 (Table 1). GRAVH analysis indicated that all ThCBM50 proteins exhibited hydrophilicity (the GRAVH value was less than zero) (Table 1). Furthermore, the prediction of subcellular localization revealed that most of the ThCBM50 proteins were located in the extracellular membrane, except for ThCBM50_2 and ThCBM50_6, which were located in the nucleus (Table 1).

### 3.2. Phylogenetic Analysis of ThCBM50 Proteins

**Figure 1:**
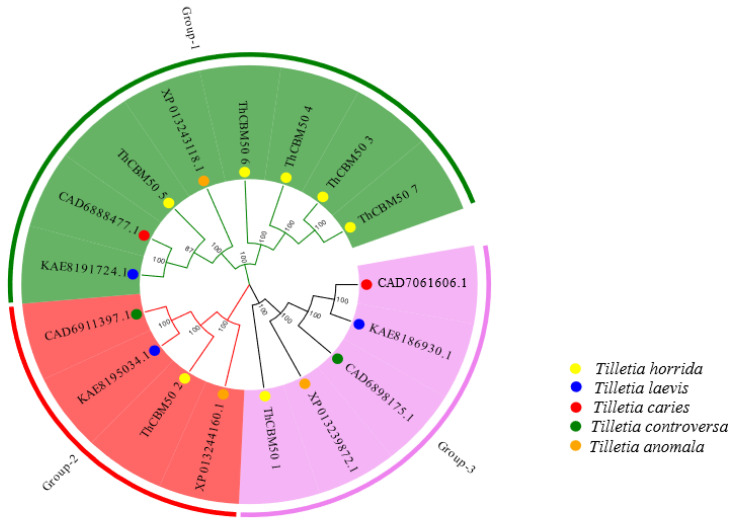
The phylogenetic trees of ThCBM50 proteins and their homologous proteins in other smut fungi, included *T. laevis*, *T. caries*, *T. controversa*, and *T. anomala*. The phylogenetic tree was constructed with MEGA7 software using the neighbor-joining (NJ) method. Different groups are illustrated as branches and frames with different colors.

To analyze the phylogenetic relationship of CBM50 family proteins in *T. horrida*, a phylogenetic tree of the seven ThCBM50 proteins and their homologous proteins in other smut fungi, including *T. laevis*, *T. caries*, *T. controversa*, and *T. anomala* was generated using MEGA7.0 software. The results revealed that these seven ThCBM50 proteins were divided into three groups, with Group I having the largest number of members encompassing five ThCBM50 proteins (Figure 1). The ThCBM50_2 and ThCBM50_1 genotypes belonged to Groups II and III, respectively (Figure 1). All the above findings indicate that the seven ThCBM50 proteins had a high homology, except for ThCBM50_2 and ThCBM50_1, which suggests that these CBM50 proteins may have similar biological functions in *T. horrida*.

### 3.3. Conserved Motifs and Gene Structure Analysis of ThCBM50 Proteins

**Figure 2:**
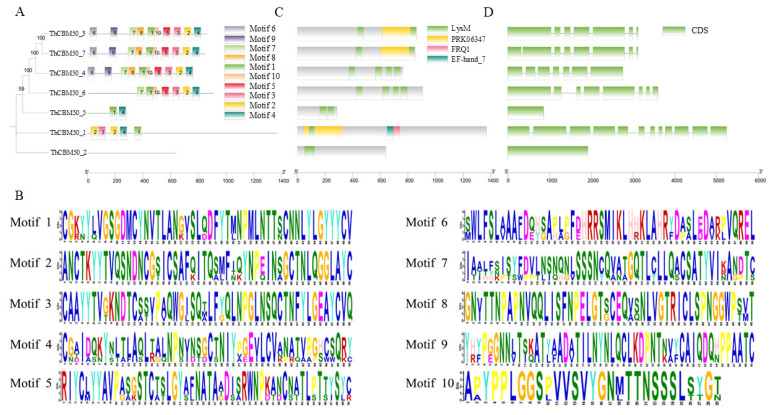
The motif distribution, conserved domain, and gene structure analysis of the seven ThCBM50 members: (**A**) the conserved motifs of the seven ThCBM50s were analyzed using the website Multiple Em for Motif Elicitation (MEME); (**B**) the conserved motif sequence logos of ThCBM50 proteins; (**C**) the conserved functional domains of the seven ThCBM50s were analyzed using the NCBI website; (**D**) exon–intron structures.

To understand the characteristics of the *T. horrida* CBM50 gene family, the conserved motifs of the seven proteins were analyzed using the website Multiple Em for Motif Elicitation (MEME). Notably, 10 different motifs in ThCBM50 proteins were identified, designated as Motifs 1–10 (Figure 2A,B). We found that the majority of ThCBM50 proteins exhibited conserved motifs, except for ThCBM50_2 (Figure 2A). A higher degree of conservation was observed in Motifs 1 and 4, presenting in six ThCBM50 proteins (Figure 2A). Furthermore, Motifs 6 and 9 were only detected in ThCBM50_3, ThCBM50_4, and ThCBM50_7, which had the highest number of conserved motifs, with all 10 motifs identified (Figure 2A). The genetic distance of ThCBM50 proteins with similar motifs was the closest, indicating that their biological function in *T. horrida* may be identical (Figure 2A). Functional domain analysis revealed that all seven ThCBM50 members have at least one LysM domain (Figure 2C). ThCBM50_4 and ThCBM50_6 had the largest number of LysM domains, with four domains (Figure 2C). The structural features of the seven genes were detected. As shown in Figure 2D, most of the ThCBM50 genes contain multiple exons and introns, except for ThCBM50_2 and ThCBM50_5, which only have one exon and no introns.

### 3.4. The Promoter Cis-Acting Element Analysis of the ThCBM50 Genes

**Figure 3:**
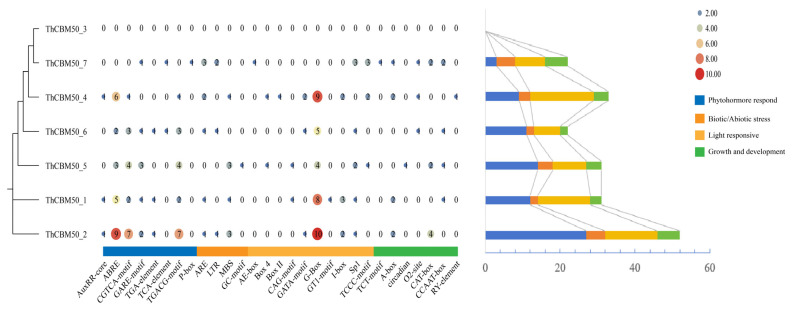
*Cis*-acting elements of ThCBM50 family members in *Tilletia horrida*. The color represents various *cis*-regulatory elements identified in the ThCBM50 genes’ promoter regions. The circle size indicates the frequency of each *cis*-regulatory element.

The effectors secreted by phytopathogens may interfere with multiple biological pathways in the host plant, facilitating pathogen infection [28,29]. Thus, to shed light on the involvement of ThCBM50 genes in the regulation of plant signaling pathways, the cis-elements in the 2000 bp sequence upstream of the seven ThCBM50 genes were analyzed. Multiple cis-elements were found in the seven genes, except for ThCBM50_3 (Figure 3 and Table S1). Hormone response elements, such as auxin-responsive, abscisic acid-responsive, MeJA-responsive, gibberellin, and salicylic acid-responsive elements, were identified in six ThCBM50 genes, suggesting that these genes may be involved in the regulation of host plant hormones during *T. horrida* infection (Figure 3 and Table S1). The biotic/abiotic elements associated with stress response, including low-temperature and anaerobic conditions, as well as the MYB binding site involved in drought inducibility, were detected in the majority of the ThCBM50 genes, indicating that they may also be involved in the regulation of host stress response (Figure 3 and Table S1). Furthermore, the light-responsive element was detected highly frequently in the ThCBM50 genes, suggesting that they may affect host plant photosynthesis (Figure 3 and Table S1). Interestingly, we found that the ThCBM50 genes contain several plant growth and development elements, such as meristem expression, indicating that these genes may influence this biological process in host plants (Figure 3 and Table S1). Thus, these results demonstrate that ThCBM50 genes are involved in the regulation of multiple biological processes in host plants, providing significant insights for the study of the biological functions of the ThCBM50 gene family in *T. horrida*–host plant interactions.

### 3.5. Protein Structure Analysis of the ThCBM50 Genes

**Figure 4:**
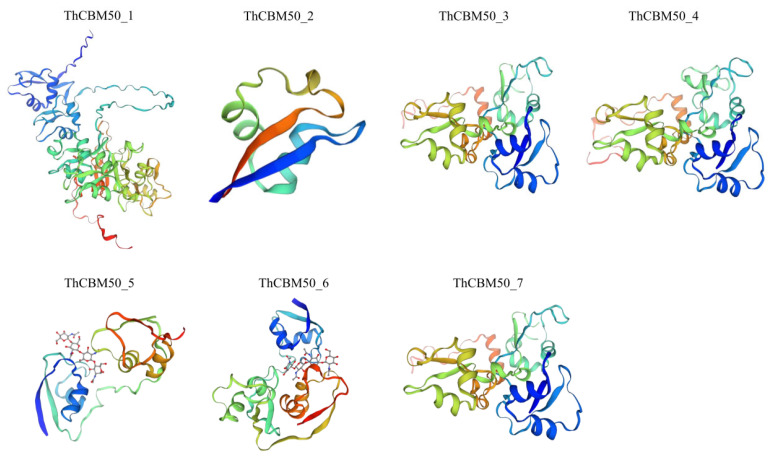
Three-dimensional (3D) modeling of ThCBM50 proteins was performed, and the results are displayed at a confidence level of >0.7.

To explain the ThCBM50 gene function in *T. horrida*, the protein structure of the seven ThCBM50 genes in *T. horrida* was analyzed. The secondary structural analysis revealed that these seven genes exhibited more alpha helices and random coils compared with beta turns and extended stands (Table S2). The proportion of alpha helices, beta turns, random coils, and extended stands ranged from 7.5% to 24.63%, 1.56% to 6.42%, 55.85% to 87.50%, and 3.44% to 14.77%, respectively (Table S2). The three-dimensional (3D) protein structures of these seven genes were further predicted (Figure 4). Interestingly, we found that the ThCBM50_3, ThCBM50_4, and ThCBM50_7 proteins share similar 3D structures, further indicating that these three ThCBM50s have the same function in *T. horrida* (Figure 4).

### 3.6. ThCBM50 Gene Response During T. horrida Infection in Rice

**Figure 5:**
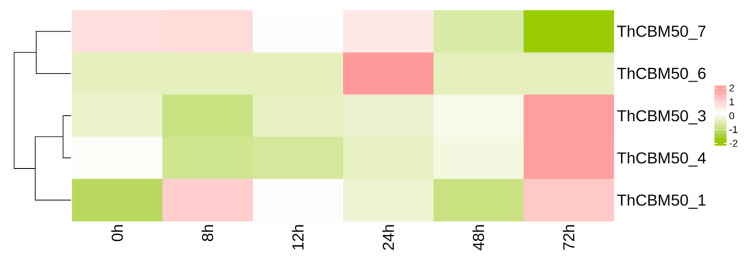
Different expression patterns of five ThCBM50 genes (FDR < 0.05 and |log2 fold change| > 1) after the inoculation of a rice variety susceptible to *T. horrida* infection (9311A). The heatmap was constructed based on the expression level of each FPKM value of the ThCBM50 genes from RNA-Seq data. Colors ranging from green to pink in the boxes indicate expression levels from lowest to highest. The digits on the scale indicate the expression levels after transcriptome data normalization.

To analyze the potential role of ThCBM50 genes in the pathogenesis of *T. horrida*, the expression pattern of the seven genes in *T. horrida* was observed after inoculation based on previously reported transcriptome data [18,19]. Of the seven genes, five exhibited different expression patterns (FDR < 0.05 and |log2 Fold Change| > 1) [18,19]. As shown in Figure 5, the expression of ThCBM50_1 was upregulated 8 h post-inoculation (hpi), and continuous upregulation was observed at 12, 24, and 72 hpi. ThCBM50_6 exhibited high expression levels at 24 hpi. Furthermore, the transcription levels of ThCBM50_3 and ThCBM50_4 exhibited significant downregulation at 8 h, followed by upregulation at 72 h. These results confirm that the expression of multiple ThCBM50 genes was induced during *T. horrida* infection, and that these genes may play a key role in the interactions between *T. horrida* and rice.

### 3.7. ThCBM50_1 Is Upregulated During T. horrida Infection

**Figure 6:**
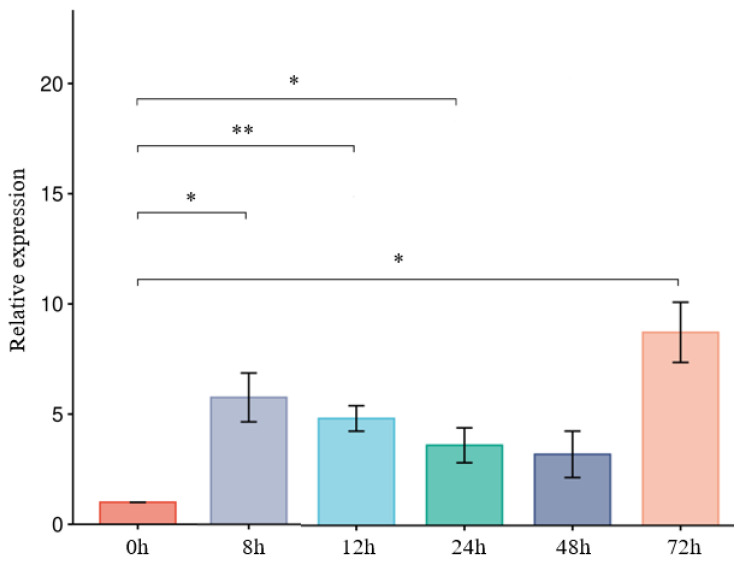
The expression of ThCBM50_1 after inoculation of a susceptible rice variety (9311A) with *T. horrida* was analyzed using qRT-PCR. UBQ expression served as an internal reference for the normalization of expression levels within the samples. Three independent experiments were performed, with four biological replicates each. The error bars indicate the standard deviation (SD) of the three independent experiments (* *p* < 0.05, ** *p* < 0.01).

The expression of effector genes in phytopathogens is typically induced at early infection stages [30,31]. According to transcriptome data, we found that the transcription levels of ThCBM50_1 were significantly upregulated at early inoculation stages. Thus, to further analyze ThCBM50_1 expression, its expression levels were observed using RT-qPCR at 8, 12, 24, 48, and 72 h after the *T. horrida* infection of the susceptible rice line 9311A. The results showed that ThCBM50_1 was continuously significantly upregulated after *T. horrida* inoculation (Figure 6), indicating that ThCBM50_1 plays an important role in *T. horrida*–rice interaction.

### 3.8. ThCBM50_1 Is Secreted Protein

**Figure 7:**
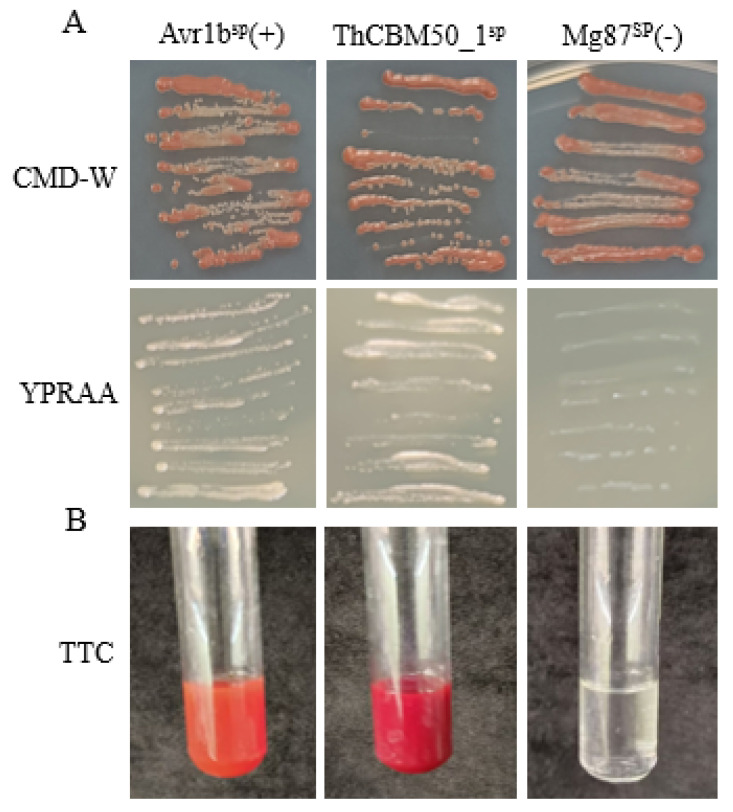
Functional analysis of ThCBM50_1′s signal peptide (SP) sequence (ThCBM50_1^SP^). (**A**) The yeast invertase secretion assay was used to identify the secretory function of ThCBM50_1^SP^. To cultivate the transformed YTK12 yeast strains, the CMD-W and YPRAA media with raffinose as the sole carbon source were each used. (**B**) The enzymatic activity of invertase was identified by performing the 2, 3, 5-triphenyltetrazolium chloride experiment. The SP sequence of *Phytophthora sojae* Avr1b was used as the positive control, and the SP sequence of *Magnaporthe oryzae* Mg87 was the negative control.

Sequence analysis revealed that the N-terminal 1–23 aa of ThCBM50_1 was a secreted protein. Thus, a yeast signal trap assay system was used to test the secretory function of ThCBM50_1^SP^ [32]. The results indicated that the fusion protein carrying ThCBM50_1^SP^, i.e., the *Phytophthora sojae* Avr1b’ SP (positive control), was found to be sufficient for the secretion of invertase in yeast YTK12, whereas the negative control did not secrete invertase in yeast (Figure 7A). The color reaction of the 2, 3, 5-triphenyltetrazolium chloride (TTC) assay was used to identify the enzyme activity of the secreted invertase, and the results revealed that the secreted invertase was able to convert the colorless TTC into insoluble red 1, 3, 5-triphenylformazan (TPF) (Figure 7B). Taken together, these results demonstrate that ThCBM50_1 is indeed a secreted protein.

### 3.9. ThCBM50_1 Induces Cell Death in N. benthamiana

**Figure 8:**
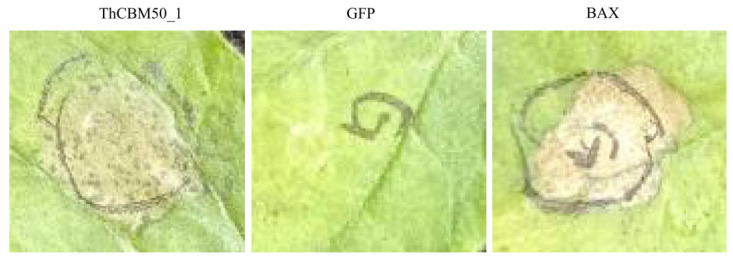
ThCBM50_1 activated the necrosis phenotype in the epidermis cells of tobacco leaves. The green fluorescent protein (GFP) served as the negative control. BAX is a mouse protein known to induce cell death in *N. benthamiana* and served as the positive control. Representative photos were taken at 4 days post-infiltration (dpi).

To investigate the ability of ThCBM50_1 to induce plant cell death, the ThCBM50_1 gene was transiently expressed in *N. benthamiana* leaves through Agrobacterium-mediated transient expression. The results revealed that ThCBM50_1 and the positive control Bax protein, known to induce cell death in *N. benthamiana*, triggered cell death in the leaves 4 days after inoculation, in contrast to the negative control green fluorescent protein (GFP) (Figure 8).

## 4. Discussion

RKS is a crucial disease that hinders the production of hybrid rice in the world [16,17]. Although previous studies have performed genome sequencing of *T. horrida* [18], and several effector proteins in this pathogen, such as ThGhd_7, ThSCSP_14, and ThSCSP_12, have been reported [27,33,34], to date, the function of CBM50 genes in *T. horrida* has remained unexplored. In this study, we comprehensively analyzed the genes belonging to the CBM50 family in *T. horrida* via a bioinformatics method. The ThCBM50 subfamily comprises seven members, all containing conserved LysM domains. Furthermore, the great majority of ThCBM50 genes have multiple conserved motifs; however, variations were observed in some gene motifs, except for ThCBM50_2. This may be due to the conservatism of ThCBM50_2 being poor, and when experiencing rapid change during evolution, obtaining results for its sequence and structure with a high degree of consistency is difficult [35].

The highly ordered structure of proteins has a direct relationship with their function [36]. Our findings showed that the ThCBM50_3, ThCBM50_4, and ThCBM50_7 proteins exhibited similar conserved motifs and 3D structures, which points to these three ThCBM50 proteins performing the same function during *T. horrida* infection. These findings allow us to preliminarily characterize the ThCBM50s and provide valuable insights for the further analysis of the role of the ThCBM50s in *T. horrida*.

It has been reported that the effector proteins secreted by phytopathogens could regulate multiple biological pathways of host plants during infection, associated with different processes such as cell wall modification, hormone synthesis, and photosynthesis [28,29]. For example, the RXLR effector PsAvh238, belonging to *Phytophthora sojae*, interferes with the ethylene (ET) signaling pathway of the host plant and facilitates pathogen infection [29]. Another *P. sojae* effector, Avh94, can hijack host plant jasmonic acid (JA) signaling to promote infection [28]. In our study, the cis-acting element analysis of the promoters of ThCBM50s revealed hormone-responsive, stress-responsive, light-responsive, and meristem expression elements in plants, indicating that ThCBM50 genes may be involved in these response processes of host plants during *T. horrida* infection.

The LysM-containing proteins are widely distributed among fungi [37,38,39,40]. The Gp15 of Bacillus phage phi 29, which contains a 1,4-beta-N-acetylmuramoylhydrolase domain and two LysM domains, is involved in morphogenesis [41]. The Rpf growth factor belonging to *Mycobacterium luteus* contains a 1,4-beta-N-acetylmuramoylhydrolase domain and a LysM domain. This gene is involved in the regulation of bacterial growth in culture via the enzymatic modification of the bacterial cell envelope [42]. The analysis of the seven identified ThCBM50 genes revealed that ThCBM50_1, ThCBM50_3, and ThCBM50_7 also contain a 1,4-beta-N-acetylmuramoylhydrolase domain (PRK06347 superfamily) and LysM domains. Thus, we speculate that these three ThCBM50 proteins may have similar biological functions to the abovementioned proteins.

Many studies have demonstrated that the CBM proteins of plant pathogens play an important role in pathogen–plant interactions [43,44]. For instance, the CMB protein PcCBP3, belonging to *Phytophthora capsici*, could trigger cell death in *N. benthamiana* leaves [7]. Furthermore, most of the fungi effectors reported thus far are secreted proteins that induce upregulation at the early inoculation stage, such as PeLysM1, PeLysM2, and PeLysM4 in *P. expansum*, which causes blue mold in pome fruit [45,46,47]. In this study, we also found that ThCBM50_1 was upregulated at 8 h after *T. horrida* infection, with an SP sequence. Thus, we used the *N. benthamiana* model system to the role of ThCBM50_1 in inducing plant cell death. These results confirmed that ThCBM50_1 serves as an effector and plays a crucial role in *T. horrida*–plant interaction. Nevertheless, to clarify the influences of ThCBM50_1 on its host, further study is needed on the stable heterologous expression of this gene in rice using genetic transformation methods. The molecular mechanism of ThCBM50 genes’ involvement in the interaction between *T. horrida* and rice also requires further exploration.

## 5. Conclusions

In this study, a comprehensive analysis of the CBM50 gene family in *T. horrida* was conducted. We analyzed the physicochemical properties, phylogenetic relationships, conserved motifs, and promoter cis-acting elements of the seven identified ThCBM50 genes. Moreover, the transcriptome data revealed that the expression of several of these genes was induced by *T. horrida* infection. Among the seven genes, ThCBM50_1 encoded a secretory protein and triggered cell death in *N. benthamiana* leaves. These findings may serve as the basis of future studies to understand the role of CBM50 proteins in *T. horrida*–host plant interactions.

## Figures and Tables

**Table 1:** The nomenclature and characteristics of the predicted carbohydrate-binding modules (CBMs) in the *T. horrida* CBM50 gene family.

Proposed Gene Name	Gene ID	Protein Length (aa)	Mw (kDa)	Instability Index	pI	GRAVH	Predicted Subcellular Localization
ThCBM50_1	smut_1650	1368	145.57	41.69	6.24	−0.25	extracellular
ThCBM50_2	smut_2305	640	65.17	52.16	10.27	−0.756	nucleus
ThCBM50_3	smut_4126	862	90.05	44.15	6.93	−0.064	extracellular
ThCBM50_4	smut_4802	763	80.01	37.02	6.84	−0.112	extracellular
ThCBM50_5	smut_5680	286	30.81	47.12	8.6	−0.466	extracellular
ThCBM50_6	smut_7273	907	98.33	57.2	8.87	−0.398	nucleus
ThCBM50_7	smut_7501	851	89.09	43.18	7.23	−0.072	extracellular

ID: identity; bp: base pair; aa: amino acids; KDa: kilodalton; pI: isoelectric point; Mw: molecular weight; GRAVH: grand average of hydropathicity.

## Data Availability

The original contributions presented in this study are included in the article; further inquiries can be directed to the corresponding authors.

## Supplementary Materials

The following supporting information can be downloaded at: https://www.mdpi.com/article/10.3390/jof10120856/s1, Table S1: *Cis*-regulatory elements are present in the ThCBM50 gene promoter regions. Table S2: Secondary structure prediction of CBM50 proteins in *T. horrida*. Table S3: The primer sequences used in this study.
